# Phytochemicals in Gynecological Cancer Prevention

**DOI:** 10.3390/ijms22031219

**Published:** 2021-01-26

**Authors:** Marta Woźniak, Rafał Krajewski, Sebastian Makuch, Siddarth Agrawal

**Affiliations:** 1 Department of Pathology, Wroclaw Medical University, 50-368 Wroclaw, Poland; marta1wozniak@wp.pl (M.W.); sebastian.mk21@gmail.com (S.M.); 2 Department and Clinic of Internal Medicine, Occupational Diseases, Hypertension and Clinical Oncology, Wroclaw Medical University, 50-556 Wroclaw, Poland; rafal.krajewski@umed.wroc.pl; 3 Department of Cancer Prevention and Therapy, Wroclaw Medical University, 50-556 Wroclaw, Poland

**Keywords:** phytochemicals, gynecological cancers, anticancer

## Abstract

Gynecological cancer confers an enormous burden among women worldwide. Accumulating evidence points to the role of phytochemicals in preventing cervical, endometrial, and ovarian cancer. Experimental studies emphasize the chemopreventive and therapeutic potential of plant-derived substances by inhibiting the early stages of carcinogenesis or improving the efficacy of traditional chemotherapeutic agents. Moreover, a number of epidemiological studies have investigated associations between a plant-based diet and cancer risk. This literature review summarizes the current knowledge on the phytochemicals with proven antitumor activity, emphasizing their effectiveness and mechanism of action in gynecological cancer.

## 1. Introduction

Currently, there is a dynamic increase in the number of cancer cases around the world. A total of 18.1 million new cases were reported in 2018, of which nearly 10 million were fatal [1]. It is estimated that a prolonged human lifespan and limited access to highly specialized anticancer treatment will result in an increase in the number of new malignant tumor cases [2]. In order to mitigate this trend, it is postulated to change people’s habits by promoting regular physical activity and a balanced diet [3]. Such an approach seems to be justified due to the fact that 90–95% of all cancer risk factors are environmental ones, of which 30–35% are dietary [4]. Epidemiological, experimental, and clinical studies are currently underway around the world to identify those components of the daily diet that may affect both the growth and reduction of cancer risk. Similar research is also being performed on gynecological tumors. In 2018, a total of 1,300,000 women worldwide suffered from gynecological cancer; and almost 610,000 cases were fatal [1]. Presented epidemiological data indicate that female genital cancers are still a serious public health concern [1]. Various first-line treatment strategies are administered based on the tumor stage and cell type, but surgery and chemotherapy are the most frequently included [5]. Despite the significant therapeutic advances in recent years, the current therapeutic options for gynecological cancers are insufficient. Novel promising targeted agents with potential anticancer effects comprise of antiangiogenic agents, poly (ADP-ribose) polymerase (PARP) inhibitors, tumor-intrinsic signaling pathway inhibitors, selective estrogen receptor downregulators, and immune checkpoint inhibitors, which target the main causes responsible for cancer development [6]. High-risk human papillomaviruses (HR-HPVs) are considered as the main etiologic factors of female lower genital tract malignancies [7]. For this reason, HPV testing is an important part of gynecological cancer screening, and immunization against HPV using vaccines has been a major step forward towards gynecological cancer prevention [8]. The most common gynecological cancers are cervical cancer, endometrial cancer, and ovarian cancer. The aforementioned cancer types are frequently characterized by mutations in K-RAS, H-RAS, BRAF, PTEN and TP53 [9,10], among others, each of which might be dietary-dependent [11]. In recent years, there has been an increasing number of studies indicating the possible anticancer effects of individual dietary components, especially those of plant origin [5]. For this reason, the authors present the results of epidemiological, experimental, and clinical studies indicating the possibility of using a plant-based diet and its components in the prevention of gynecological cancer. The fundamental goal of this review was to discuss the clinical significance and possible benefits of phytochemicals in the chemoprevention of female reproductive system cancers.

## 2. Chemoprevention

Chemoprevention uses synthetic, natural, or biological chemicals in order to inhibit, delay, or reverse carcinogenesis [12]. The preventive method was first introduced by Sporn et al. [13] and among others, uses compounds derived from commonly used vegetables, fruit, and whole-grain products—so-called phytochemicals, which are chemically divided into polyphenols, terpenoids, and thiols (Table 1) [14,15]. Chemoprevention may play an important role in reducing the development of various types of cancer, as nearly 33% of them can be prevented by lifestyle changes, including diet [14].

A model chemopreventive compound is one that is safe to use, has a known mechanism of action, can be administered orally, and is characterized by high efficiency and a low production cost [105]. Thanks to such features, it is possible to use it widely in the prevention of many diseases, including cancer [106,107,108]. In vitro and in vivo studies have shown a wide range of anticancerogenic effects of phytochemicals. Some of them have an anti-inflammatory effect by limiting the secretion of cytokines by cells, e.g., IL–1, IL–6, IL–8, IL–12, TNF–alfa [109]. In turn, by inhibiting VEGFA or PI3/Akt, they prevent cells from developing angiogenesis, which determines the growth and further development of cancer [110]. What is more, most phytochemicals act as antioxidants, which by eliminating free oxygen radicals, protects the cell’s genetic material from damage and possible carcinogenesis [111]. In addition to reducing the risk of cancer, compounds of plant origin can also influence its treatment by improving both the sensitivity of tumors to chemotherapeutics and their demonstrated in vitro cytotoxic effects [112,113].

**Figure 1:**
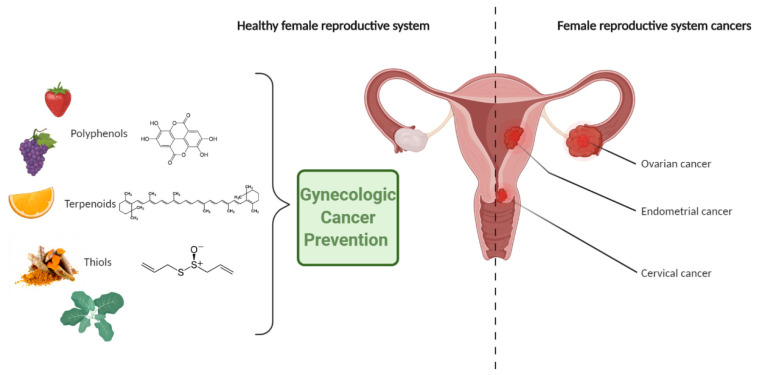
Polyphenols, terpenoids and thiols in gynecological cancer prevention.

Due to the experimentally proven anticancerogenic effects of the selected phytochemicals, research is currently underway to assess their possible use in cancer prevention—including gynecological cancers. The further part of the paper presents and discusses phytochemicals, which, in our opinion, are the most crucial in the chemoprevention of the most common malignant tumors of female genital organs, i.e., cervical cancer, endometrial cancer, and ovarian cancer (Figure 1).

## 3. Phytochemicals in Cervical Cancer Prevention

In the GLOBOCAN 2018 study, cervical cancer was ranked fourth in terms of both incidence (569,847 cases) and mortality (311,365 cases) from all cancers in the female population [1]. Its origin and development are strongly linked to the occurrence of the persistent infection of oncogenic strains of human papilloma virus (HPV) [114], in particular, HPV-16 and HPV-18 [21]. It is a DNA virus whose global prevalence is estimated at 11.7% [115]. Cytology, vaccination, and sex education are essential factors in limiting its spread in the population [116] and thus also reduce incidence and mortality due to cervical cancer [117]. Currently, more and more attention is also being paid to the possible influence of diet on the development of presented cancer [118,119]. Due to its anticancer action, plant-based compounds can play a special role in the prevention of various cancers, including cervical cancer [120].

### 3.1. Polyphenols

Numerous in vivo and/or in vitro studies indicate the anticancer effect of polyphenols [121], which is primarily based on the induction of apoptosis through various intracellular pathways. Convercetin—a flavonoid of onions, red grapes, broccoli, and apples—acts by increasing the concentration of the p53 protein and inhibiting the NF-κB pathway. [16]. Fisetin—a strawberry and cucumber flavonoid—activates enzymes from the caspase family 3 and 9 [122]. Naringin—grapefruit flavonoid—leads to cell death by the induction of death receptors [33]. Other mechanisms of action of polyphenols include the scavenging of free oxygen radicals, limiting cell proliferation, and reducing inflammation [123]. Experimental studies have shown that the following phytochemicals may be relevant among polyphenols in the chemoprevention of cervical cancer: kaempferol [21], rutin [24], gallic acid [49], ferulic acid [55], epigallocatechin gallate (EGCG) [39], curcumin [60], and caffeic acid [56]. This indicates significant potential for polyphenols in the chemoprevention of cervical cancer. Nevertheless, despite numerous experimental studies, there are currently few epidemiological studies available in the literature to verify their results. In an extensive cohort study, Gonzalez et al. investigated the correlation between different dietary factors and among others, the risk of invasive cervical cancer. It was observed that the consumption of fruit at a dose of 100 g/day was associated with a statistically significant reduction in the risk of invasive cervical cancer (HR = 0.83, 95% CI = 0.72–0.98). A similar relation was noted for vegetables, although it did not prove statistically significant (HR = 0.85, 95% CI = 0.65–1.10) [124]. In addition, the meta-analysis of Tomita et al. (a total of 18 studies: 17 clinical control studies, one cohort study) showed that among women eating more fruit and vegetables, cervical cancer was 20 and 40% less frequent, respectively [125]. In the study by Barchitta et al., the authors showed that moderate adherence to the Mediterranean diet could reduce the risk of HPV infection compared to low adherence to it (OR = 0.40, 95% CI = 0.22–0.73) [126]. The results of the study indicate the possibility of preventing cervical cancer by reducing the risk of infection with an oncogenic HPV strain.

### 3.2. Terpenoides and Thioles

Terpenoids include carotenoids, whose anticancerogenic value has been confirmed by experimental studies [127]. It seems that they may also be important in preventing the development of cervical cancer. Zhang et al. studied the relationship between the plasma concentrations of carotenoids, retinol, and tocopherol and the risk of cervical cancer among Chinese women. They proved that higher concentrations of carotenoids (alpha-carotene, beta-carotene, lutein, zeaxanthin) and tocopherol (alpha-tocopherol) were associated with a lower risk of cervical cancer (OR = 0.71, 95% CI = 0.56–0.92, *p* = 0.003; OR = 0.75, 95% CI = 0.60–0.94, *p* = 0.008 respectively). In the same study, the authors demonstrated a statistically significant effect of individual carotenoids in reducing this risk for: alpha-carotenes OR = 0.50, 95% CI = 0.38–0.67, *p* < 0.001; beta-carotenes OR = 0.70, 95% CI = 0.56–0.88, *p* = 0.002; lutein and zeaxanthin OR = 0.75, 95% CI = 0.59–0.95, *p* = 0.015 [71]. Another compound from the group of terpenoids exhibiting anticancer effects is lycopene, which is found in tomatoes [128]. For cervical cancer cells, in vitro studies have shown that it can increase their sensitivity to cisplatin [74]. Other terpenoids with possible anticancer effects are ursolic acid [86] and ginkgolide B [90]. However, the results have not yet been confirmed by epidemiological studies. Among thiols, indole-3-carbinol (I3C) seems to be a promising chemopreventive compound, which can mainly be found in broccoli and Brussels sprouts. In vitro studies on cervical cancer cells have shown its anticancer effects [98]. The results were confirmed by a randomized, double-blind, multi-center clinical trial with a placebo assessing the effects and safety of 3,3′-diindolymethane (DIM, a stable form of I3C) for the treatment of cervical intraepithelial neoplasia (CIN). A total of 78 patients aged 18-39 years (56 out of 78 diagnosed with CIN I or CIN II) were included in this study. The research group applied DIM in the form of vaginal suppositories for 180 days. In the group of women receiving DIM at a dose of 200 mg/day and 100 mg/day, a 100% (CI 95% = 82.35–100.00%) and 90.5% (CI 95% = 69.62–98.83%) regression of CIN lesions on histological examination were observed, respectively. However, in the placebo group, only 61.1% of the patients had a regression of the lesions on histological examination (CI 95% = 35.75–82.70%) [129]. The presented results indicate a significant potential of I3C and its DIM derivative in the chemoprevention of cervical cancer. The allicin present in garlic and onion may also have an important preventive function for the analyzed type of cancer. In vitro studies have shown that reducing the expression of nuclear factor erythroid 2-related factor 2 leads to the death of cervical cancer cells [95].

## 4. Phytochemicals in Endometrial Cancer Prevention

In 2018, 382,096 new cases of endometrial cancer were reported worldwide, of which nearly 90,000 were fatal [1]. In terms of incidence, endometrial cancer ranks sixth among all female malignant cancers [1], and currently, the number of new cases is systematically increasing. This may be related to the obesity epidemic, which has doubled worldwide in less than 30 years [130]. This, in turn, is confirmed by the fact that 34% of all cases of endometrial cancer are related to a high body mass index (BMI) [130]. Compared to women with a BMI < 25 kg/m^2^, women with BMI > 30 kg/m^2^, BMI < 35 kg/m^2^, and with BMI > 35 kg/m^2^ had a 2.6- and 4.7-times higher risk of developing the disease, respectively [131]. This phenomenon may be explained, among others, by the high estrogen content in adipose tissue, which promotes the growth of the uterine mucosa [132]. Moreover, adipose tissue is the place of the secretion of adipokines, i.e., compounds of pro-inflammatory nature (e.g., IL-6, leptin) having the ability to induce pathways leading to consequent carcinogenesis. [132]. Obesity may be the consequence of an unbalanced diet, which in a two-fold way influences the risk of developing the analyzed cancer; a diet high in animal products may increase the risk of disease [133], while a diet rich in vegetables, fruit, or fish can reduce its risk [134]. The limiting effect of diet—especially a plant-based diet—on the development of endometrial cancer may be related to the anticancer effect of its individual components, including phytochemicals.

### 4.1. Polyphenols 

Polyphenols include flavonoids—chemical compounds commonly found in vegetables and fruits—and their representatives include isoflavones such as genistein, daidzein, and glycitein [15]. It has been suggested that high doses of isoflavones show anti-estrogenic effects preventing the development of endometrial cancer [135]. A meta-analysis by Zhong et al. investigated the relationship between the consumption of isoflavones contained in soybean and legumes and the risk of endometrial cancer. The research included a total of 13 studies—3 cohort studies and 10 clinical control studies. The researchers found that compared to a lower isoflavone intake, its higher intake was associated with a 19% reduction in the risk of endometrial cancer (OR = 0.81, 95% CI = 0.74–0.89). [136]. Such a reduced risk was also noted in the meta-analysis by Grosso et al. [137]. The possible chemopreventive effect of isoflavones was also evidenced by a cohort study by Ollberding et al., in which the authors showed that the consumption of isoflavones by women in the postmenopausal period was associated with a statistically significant 34% reduction in the risk of developing the disease (RR = 0.66, 95% CI = 0.47–0.91, *p* = 0.02), while the consumption of individual isoflavone components, such as daidzein or genistein, was also associated with a statistically significant reduction in this risk by 36% (RR = 0.64, 95% CI = 0.46–0.90, *p* = 0.01) and 34% (RR = 0.66, 95% CI = 0.47–0.91, *p* = 0.02), respectively [30]. In the same study, the authors estimated that in the research group of women, the risk of endometrial cancer can be reduced by 27% with a dose of isoflavones amounting to at least 7.82 mg per 1000 kcal per day. Isoflavone intake can reduce the growth of uterine mucosa and thus reduce the risk of developing endometrial cancer. The relationship between daily isoflavone intake and its effect on endometrial thickness was the subject of a meta-analysis of 23 randomized clinical trials by Li et al., who showed that daily isoflavone supplementation at a dose greater than 54 mg can reduce endometrial thickness by 0.26 mm in postmenopausal women (*p* = 0.007). Moreover, not all studies confirm the above dependence [138]. In addition to isoflavones, other compounds from the polyphenol group seem to be important in the chemoprevention of endometrial cancer. Such a compound is an ingredient of green tea—epigallocatechin gallate [15], which has a comprehensive anticancer effect [139]. One of them is to prevent angiogenesis by reducing the concentration of vascular endothelial growth factor A (VEGFA), which has been proven by both in vivo and in vitro studies on endometrial cancer cell lines [40]. This can be confirmed by a meta-analysis by Zhou et al. evaluating the correlation between the consumption of green and black tea and the risk of endometrial cancer. It has been proven that the daily consumption of one cup of green tea was associated with an 11% lower risk of developing endometrial cancer (RR = 0.89, 95% CI = 0.84–0.94). Nevertheless, a similar relationship has not been demonstrated to this extent for black tea (RR = 0.99, 95% CI = 0.94–1.03) [140]. Similar conclusions were also drawn in the meta-analysis by Butler et al. in which the researchers observed that the consumption of green tea was associated with a 23% reduction in the risk of endometrial cancer (OR = 0.78, 95% CI = 0.62–0.98) [141]. Although green tea owes its potential anticancer effect to various catechins, it seems that the analyzed epigallocatechin gallate, which accounts for 50-70% of their concentration, is largely responsible for this effect [142]. However, it is worth mentioning that some meta-analyses have not shown the relationship between tea consumption and endometrial cancer risk [143,144]. For this reason, other polyphenolic compounds with promising chemopreventive potential in endometrial cancer are still being sought. Current in vitro studies have shown that some of them (quercetin [18], kaempferol [22], resveratrol [145], and hesperidin [36]) inhibit the development of carcinogenesis by affecting intracellular transmission. The presented studies indicate the significant chemopreventive potential of polyphenols in endometrial cancer. This is also confirmed by a clinical control study by Gifkins et al., which showed that the consumption of products containing polyphenolic compounds reduces the risk of developing endometrial cancer (OR = 0.62; 95% CI = 0.39–0.98) [146].

### 4.2. Terpenoids and Thiols

The Mediterranean diet (MD) largely consists of fruit and vegetables rich in phytochemicals belonging to terpenoids and thiols—carotene, lutein, zeaxanthin, lycopene, astaxanthin, phytosterols, or isothiocyanates [147,148]. As mentioned above, all of them may exhibit anticancer effects to varying degrees. For this reason, research is underway to assess the impact of the Mediterranean diet on cancer development, including endometrial cancer. One of them was a clinical control study by Ricceri et al. in which the authors showed that higher vegetable consumption, following the Mediterranean diet, which has a low content of pro-inflammatory compounds which is associated with a lower risk of developing endometrial cancer (respectively: OR fifth quintile vs. first quintile = 0.34, 95% CI = 0.17–0.68; OR = 0.51, 95% CI = 0.39–0.86; OR fifth quintile vs. first quintile = 3.28, 95% CI = 1.30–8.26) [93]. Similar results were also obtained by Filomeno et al. (OR for high adhesion MD = 0.43, 95% CI = 0.34–0.56) [134]. Nevertheless, in recent years, there have been several experimental studies exploring new directions in the search for effective phytochemicals in endometrial cancer chemoprevention. The first example is asparanin A, which is a saponin found in medical asparagus (Asparagus officinalis L). The plant contains numerous compounds showing various effects, including anticancerogenic ones [149]. Zhang et al. were the first to demonstrate in vitro on the Ishikawa cell line that asparanin A can limit the proliferation of endometrial cancer cells and induce their apoptosis [80]. Another example is ginsenosides [150], which are found in ginseng root (Panax ginseng C. A. Meyer). Jo et al. have shown in vitro on the cell line (HEC)-1A that 20(S)-protopanaxadiol (one of the ginsenosides) leads to the death of endometrial cancer cells by the induction of apoptosis [151]. Despite the promising results of experimental studies, more research is still necessary, especially on the epidemiological type, which would verify the presented results and assess their possible chemopreventive effects on endometrial cancer. In turn, the literature currently devotes much attention to coffee—the most widely consumed beverage in the world [152,153], which owes its biological action to various compounds, such as caffeine, chlorogenic acid, cafestol, and kahweol. The last two compounds—cafestol, kahweol—belong to terpenoids [152,153], and it is to a large extent to these compounds that coffee owes its anticancerogenic properties [154], which have been confirmed by numerous studies assessing the relationship between coffee consumption and the risk of developing endometrial cancer. A meta-analysis of Lukic et al. included 12 cohort studies and eight clinical control studies. The authors observed that, compared to lower coffee intake, its higher level was connected with endometrial cancer prevention (total RR = 0.74, 95% CI = 0.68–0.81; for cohort studies RR = 0.78, 95% CI = 0.71–0.85; for clinical control studies RR = 0.63, 95% CI = 0.53–0.76). In the same research, it was estimated that in cohort studies, drinking one cup of coffee per day was associated with a 3% reduction in the risk of endometrial cancer (95% CI = 2–4%), and in clinical control studies with a 12% reduction (95% CI = 5–18%) [57]. Similar conclusions were reached by the Lafranconi et al., who reported a 20% reduction in the risk of the disease with the consumption of four and more cups of coffee a day (RR = 0.80, 95% CI = 0.72–0.89) and 24% for postmenopausal cancers (RR = 0.76, 95% CI = 0.69–0.83) [58]. A reversal relationship between coffee consumption and the risk of endometrial cancer was also noted by Poole et al. [152].

## 5. Phytochemicals in Ovarian Cancer Prevention

The GLOBOCAN 2018 study ranks ovarian cancer as fourth in terms of incidence and third in terms of mortality among female genital malignant tumors [1]. Recent epidemiological data indicate a downward trend in morbidity and mortality due to ovarian cancer [155], which is probably due to the use of oral hormonal contraception—a recognized preventive factor for ovarian cancer [156,157,158]. Nevertheless, despite this seemingly optimistic trend, ovarian cancer still poses a great danger for women, as currently there are no effective screening tests that would allow for its early detection and treatment while improving the survival of this group of patients [159]. For this reason, the majority of patients are diagnosed too late when the disease is already in its advanced stage, and 5-year survival in such a situation decreases even to 29% [160]. In addition, the effectiveness of the standard treatment of ovarian cancer, including surgical procedures with subsequent chemotherapy, depends on many factors, including the severity of the tumor and its molecular profile [161,162], which undoubtedly have a significant impact on the further prognosis of the patients. As in the case of cervical and endometrial cancer, a possible relationship between the type of diet and the risk of its development is currently being sought in ovarian cancer [163,164,165]. Phytochemicals present in plants may play an important role in reducing the risk of ovarian cancer due to their anticancer properties [166,167,168].

### 5.1. Polyphenols

Among the polyphenols, curcumin, found in turmeric, seems to be a promising chemical compound. Kuttan et al. first demonstrated the anticancerogenic properties of this compound in an experimental study in 1985 [169,170]. Since then, intensive research on its possible use in medicine, including oncology, has begun. Currently, there are no epidemiological studies that would allow to assess the effect of curcumin on ovarian cancer chemoprevention in the human population. However, the results of experimental studies indicate the possibility of its use in improving the treatment of patients with this type of cancer, as curcumin, through its anti-inflammatory and antioxidant effects, can limit the process of carcinogenesis [171,172]. In addition, a study by Seo et al. showed that curcumin, by inhibiting the sarco/endoplasmic reticulum calcium ATPase (SERCA) proteins, causes a disturbance in the calcium homeostasis of tumor cells, thus contributing to the induction of their apoptosis [62]. Moreover, the studies indicate that curcumin may be used as a means to reduce the drug resistance of ovarian cancer cells [173,174]. However, despite the experimental demonstration of the importance of curcumin in the treatment of ovarian cancer, its use is currently limited due to the lack of clear results from human studies [175]. Another compound from the group of polyphenols, which, due to its mechanisms of action, can be used in the chemoprevention of ovarian cancer, is resveratrol. The main source of this phytochemical is grape peel and wine [15]. The anticancer action of resveratrol consists not only of the induction of apoptosis but also of the autophagocytosis of ovarian cancer cells [66,67,176]. In addition, in vivo mouse studies showed that after applying cisplatin for 48 hours and then resveratrol for 72 hours, the frequency of ovarian cancer cell proliferation was significantly reduced [67]. An interesting mechanism of resveratrol action is the inhibition of the GLUT1 glucose transporters and the restriction of glucose introduction into cancer cells, which limits their growth and directs them to the apoptosis pathway [145]. Despite the promising results of the experimental studies, the epidemiological studies to date do not confirm them. A meta-analysis by Kim et al. assessed the relationship between wine consumption and ovarian cancer risk. An analysis of 10 studies (three cohort studies and seven clinical control studies) did not show the association between wine consumption and a reduction in the risk of ovarian cancer (OR = 1.13, 95% CI = 0.92–1.38) [177]. However, the insufficient number of epidemiological studies assessing the effect of resveratrol on the risk of ovarian cancer does not allow to draw clear conclusions. Therefore, more such research is needed to confirm the results obtained from experimental studies. In recent years, the attention of scientists has been drawn to the catechins contained in green tea, and particularly their representative—epigallocatechin gallate. Catechins are one of the more researched groups of chemicals in cancer prevention, including ovarian cancer. A meta-analysis of seven cohort studies and 11 clinical control studies showed a statistically significant reduction in the risk of ovarian cancer among women with family history and tea consumption. (RR = 0.86, 95% CI = 0.76–0.96) [178]. Similar conclusions were obtained in the meta-analysis of Gao et al. (OR = 0.81, 95% CI = 0.73–0.89, *p* < 0.0001) [179]. It is worth mentioning that numerous epidemiological studies have shown a positive correlation between the consumption of green tea and the reduction in ovarian cancer risk [141,180,181]. The above is also confirmed by the results of experimental research indicating that inhibiting the pathway of transforming growth factor-beta (TGF-beta) catechins may reduce the ability of tumor cells to metastasize [182].

### 5.2. Terpenoids

Carotenoids are a group of compounds that originate from carrots, tomatoes, peaches, mangoes, broccoli, spinach, or lettuce. The most important representatives of this group are alpha- and beta-carotenes and lycopene [15]. Similar to other plant-based compounds, they may exhibit anticancerogenic effects against many types of cancers: cervical cancer [183], endometrial cancer [184], and breast cancer [185]. Currently, data on the use of alpha- and beta-carotenes in the chemoprevention (or even treatment) of ovarian cancer are inconclusive. On the one hand, an older study based on a meta-analysis showed a 16% reduction in the risk of developing ovarian cancer in the case of an increased consumption of beta-carotene-rich products (RR = 0.84, 95% CI = 0.75–0.94) [72]. This can be confirmed by an experimental study by de Souz et al., who demonstrated in vitro that tropical Brazilian fruits such as Murici and tapereba containing, among others, alpha- and beta-carotenes, may have cytotoxic properties against ovarian cancer cells (including those resistant to platinum derivatives) manifesting themselves as cell cycle inhibition and the induction of their apoptosis [186]. On the other hand, a randomized analysis carried out by Guo et al. showed that beta-carotene may increase the risk of epithelial ovarian cancer, but it may also reduce the risk of developing tumors with a low malignant potential [187]. In view of the above, further research is needed to determine the clear effect of carotenoids on the development of ovarian cancer. A meta-analysis of Li et al. showed a 3.7% reduction in the risk of developing ovarian cancer if women followed a diet including lycopene in the postmenopausal period (OR = 0.963, 95% CI = 0.859–1.080), although this relationship did not prove statistically significant [75]. However, experimental research provides promising results. Holzapfel et al. demonstrated in an in vivo study that lycopene can reduce the risk of ovarian cancer cell metastasis [76]. Another study has shown that in vitro lycopene, by increasing the concentration of Bax pro-apoptotic proteins and decreasing the concentration of Bcl2 antiapoptotic proteins, induced programmed ovarian cancer cell death [77]. In turn, lycopene applied at a dose of 200 mg/kg and 400 mg/kg significantly reduced the risk of ovarian cancer in vivo in laying hens (*p* < 0.010) [188]. It is also worth noting that the European Food Safety Authority has confirmed the safety of lycopene, which is now commonly used as a preservative [189]. The clinical significance of carotenoids may be demonstrated by the Eid et al. study, which investigated the effect of adding fucoxanthins (carotenoids) to anticancer treatment on the reversal of the drug resistance of various types of cancer, including ovarian cancer. Their results showed a significant correlation between the combination of fucoxanthin and doxorubicin (anticancer drug) and the reduction in cancer cell resistance to the applied treatment [73].

### 5.3. Thiols

Glucosinolates comprise a group of chemical compounds whose highest concentration can be found in cruciferous vegetables: white cabbage, red cabbage, savoy cabbage, and napa cabbage. The representatives of this group are thiocyanates, isothiocyanates, nitriles, and indoles [15]. Chemopreventive properties have been demonstrated in particular for isothiocyanates (phenylethyl isothiocyanate, PEITC) and indoles (indole–3–carbinol, I3C) [190]. Glucosinolates have an anticarcinogenic effect on many types of cancers [191], including lung cancer [192], endometrial cancer [193], and colorectal cancer [194]. With regard to ovarian cancer, scientific data indicate the promising potential of PEITC. A meta-analysis by Jiyi et al. showed a positive correlation between the consumption of cruciferous vegetables and a reduced risk of ovarian cancer (RR = 0.89, 95% CI = 0.81–0.99) [195]. Similar conclusions were drawn from a meta-analysis conducted by Chinese researchers, in which the analysis of clinical control studies showed a reduced risk of ovarian cancer when following a diet rich in cruciferous vegetables (RR = 0.84, 95% CI = 0.75–0.94). However, no similar relationship was found in cohort studies (RR = 1.00, 95% CI = 0.85–1.11) [196]. In turn, experimental studies have shown that PEITC caused the apoptosis of ovarian cancer cells by inducing the formation of reactive oxygen species in such cells [197]. In another PEITC study, by blocking the mTOR–STAT3 pathway, PEITC reduced the ability of cancer cells to metastasize [94]. I turn, in the case of the simultaneous administration of PEITC and metformin, an increase in the sensitivity of cancer cells to cisplatin was observed [198]. This discovery may be important in the treatment of chemically resistant forms of ovarian cancer. Due to its molecular mechanisms of action, PEITC seems to be applicable not only in chemoprevention but also in the treatment of ovarian cancer. It is also noteworthy that in vitro and in vivo studies showed a good tolerance of healthy cells to the used phytochemical compound [199]. In the case of I3C, experimental studies have shown that through its active metabolites (3,3’-diindolylmethane), it blocks the epidermal growth factor receptor (EGFR), whose overexpression is observed in ovarian cancer, among others. The aforementioned inhibition leads to the intracellular blocking of signals allowing the cells to proliferate and invade [99,200]. This compound may also be important in limiting the growth of ovarian cancer [201]. Significantly, the addition of I3C and/or epigallocatechin gallate to the standard therapy (surgical treatment, taxa) of advanced stage III or IV ovarian cancer was associated with both an increase in the overall survival of the patients and a period free of disease progression compared to the absence of these compounds in therapy [202]. Currently, there are no epidemiological data confirming the experimentally proven effect of I3C in the prevention of ovarian cancer.

## 6. Conclusions

The anticancerogenic effect of phytochemicals on the development of gynecological cancers is currently the subject of many scientific studies (Table 1). Experimental studies carried out in vitro and in vivo indicate their significant potential to reduce the development of cervical, endometrial as well as ovarian cancer. Moreover, the results presented in the paper indicate that phytochemicals can exert their chemopreventive effects in two ways. On the one hand, they reduce the risk of cancer development by inhibiting the early stages of carcinogenesis, i.e., initiation and promotion. On the other hand, when included in standard chemotherapy, they increase the sensitivity of cancer cells to its effects and thus may limit the progression of cancer. However, epidemiological studies (clinical control and cohort studies) do not always confirm the results presented in experimental studies. The reason for this may be the applied dose of phytochemicals, their variable assimilability, varied metabolism, or a mechanism of action different from those presented in experimental studies. Additionally, it should be mentioned that apart from the benefits, there may also be several limitations associated with the use of phytochemicals, such as: 

- Their ambiguous effect on chemoprevention; 

- The lack of data indicating the optimal and toxic doses; 

- The lack of data regarding their potential side effects;

- The lack of data evaluating their pharmacodynamic properties;

- Contradictory results regarding the molecular pathways related to their action.

Moreover, most epidemiological studies assess the overall impact of fruit and vegetable consumption on the development of various types of cancer. Plants are the source of many phytochemicals, and for this reason, it is not always possible to clearly determine which chemical compound has a significant anticancer effect. Nevertheless, it seems that phytochemicals have a significant preventive as well as therapeutic potential regarding gynecological cancers. For this reason, the consumption of larger quantities of vegetables, fruit, and whole-grain products should be a recommended action for the prevention of cancers, including gynecological ones.

## Figures and Tables

**Table 1:** Phytochemicals in gynecological cancer prevention: classification, natural occurrence, and application.

Phytochemicals	Classification			Natural Occurrence	Application with References		
	Group	Subgroup	Class		Cervical Cancer	Endometrial Cancer	Ovarian Cancer
Quercetin	Polyphenols	Flavonoids	Flavonols	Onion, kale, leek, broccoli, buckwheat, red grapes, tea, apples	[16,17]	[18]	[19,20]
Kaempferol	Polyphenols	Flavonoids	Flavonols		[21]	[22]	[23]
Rutin	Polyphenols	Flavonoids	Flavonols	Cpers, olives, buckwheat, asparagus	[24]	ND	ND
Apigenin	Polyphenols	Flavonoids	Flavones	Clery, herbs, parsley, chamomile, rooibos tea, capsicum pepper	[25]	[26]	[27]
Luteolin	Polyphenols	Flavonoids	Flavones		[17]	ND	[28]
Genistein	Polyphenols	Flavonoids	Isoflavones	Soya, beans, chickpeas, alfalfa, peanuts	[29]	[30]	[31]
Daidzein	Polyphenols	Flavonoids	Isoflavones		ND	[30]	[32]
Naringenin	Polyphenols	Flavonoids	Flavanones	Citrus fruit	[33]	[34]	ND
Hesperitin	Polyphenols	Flavonoids	Flavanones		[35]	[36]	[37]
Anthocyanidins	Polyphenols	Flavonoids	-	Red grapes, blueberries, cherries, strawberries, blackberries, raspberries	[38]	ND	ND
Epigallocatechin gallate (EGCG)	Polyphenols	Flavonoids	Flavan–3–ols tannins	Tea, chocolate, grapes	[39]	[40,41]	[42,43]
Silymarin	Polyphenols	Flavonoids	Flavanolols	Milk thistle, red onions	[44]	ND	[45]
Silibinin	Polyphenols	Flavonoids	Flavanolols		[46]	[47]	[48]
Gallic acid	Polyphenols	Phenolic acids	Hydrobenzoic acids	Blackberries, grape seed, pomegranate, raspberries, tea, vanilla	[49]	ND	[50]
Ellagic acid	Polyphenols	Phenolic acids	Hydrobenzoic acids		[51,52]	[53]	[54]
Vanillic acid	Polyphenols	Phenolic acids	Hydrobenzoic acids		ND	[53]	ND
Ferulic acid	Polyphenols	Phenolic acids	Hydroxycinnamic acids	Blueberries, cinnamon, coffee, kiwi fruit, plums, wheat bran	[55]	ND	ND
Caffeic acid	Polyphenols	Phenolic acids	Hydroxycinnamic acids		[56]	[57,58]	[59]
Curcumin	Polyphenols	Non-flavonoid polyphenols	Curcuminoids	Turmeric	[51,60]	[61]	[62]
Cinnamic acid	Polyphenols	Non-flavonoid polyphenols	Stilbenes	Blueberries, grapes, peanuts, raspberries, wine	[63]	ND	ND
Resveratrol	Polyphenols	Non-flavonoid polyphenols	Stilbenes		[64]	[65]	[66,67]
Enterolactone	Polyphenols	Non-flavonoid polyphenols	Lignans	Grains, flaxseed, sesame seeds	ND	[68]	[69]
Sesamin	Polyphenols	Non-flavonoid polyphenols	Lignans		[70]	ND	ND
+Alpha–, beta–, gamma–carotene	Terpenoids	Carotenoid terpenoids	-	Carrots, kale, pumpkin, sweet potato	[71]	ND	[72,73]
Zeaxanthin	Terpenoids	Carotenoid terpenoids	-	Corn, eggs, kale, spinach, red pepper, pumpkin, oranges	[71]	ND	ND
Lycopene	Terpenoids	Carotenoid terpenoids	-	Tomatoes watermelon, pink grapefruit, guava, papaya	[71,74]	ND	[75,76,77]
Astaxanthin	Terpenoids	Carotenoid terpenoids	-	Salmon, shrimp, krill, crab	ND	ND	[78]
Saponins	Terpenoids	Non-carotenoid terpenoids	-	Chickpeas, soya beans	[79]	[80]	[81]
Perillyl alcohol	Terpenoids	Non-carotenoid terpenoids	-	Caraway seeds, cherries, mint	ND	ND	[82]
Siosterol	Terpenoids	Non-carotenoid terpenoids	Phytosterols	Vegetable oils, cereal grains, nuts, shoots, seeds and their oils, whole grains, legumes	[83]	ND	ND
Stigmasterol	Terpenoids	Non-carotenoid terpenoids	Phytosterols		ND	[84]	[85]
Ursolic acid	Terpenoids	Non-carotenoid terpenoids	-	Apples, cranberries, peppermint, prunes, oregano, thyme	[86]	[87]	[88,89]
Ginkgolide and bilobalide	Terpenoids	Non-carotenoid terpenoids	-	Ginkgo biloba	[90]	ND	[91]
Isothiocyanates	Thiols	-	Glucosinolates	Cruciferous vegetables such as asparagus, broccoli, Brussel sprouts, cauliflower, horseradish, mustard, radish, sprouts	[92]	[93]	[94]
Allicin	Thiols	-	Allylic sulfides	Garlic, leeks, onions	[95]	[96]	[97]
Indole–3–carbinol (I3C)	Thiols	-	Indoles	Broccoli, brussel, sprouts	[98]	ND	[99]
Betaines	Others	-	-	Beetroot	[100]	ND	ND
Capsaicin	Others	-	-	Chili	ND	ND	[101]
Piperine	Others	-	-	Black peppers	[102]	ND	[103,104]

ND—no data.

## Data Availability

Not applicable.

## Abbreviations

VEGFA	Vascular endothelial growth factor A
HPV	Human papilloma virus
EGCG	Epigallocatechin gallate
CIN	Cervical intraepithelial neoplasia
DIM	3,3‘-diindolymethane
SERCA	Sarco/endoplasmic reticulum calcium ATPase
TGF-beta	transforming growth factor-beta
PEITC	phenylethyl isothiocyanate
EGFR	Epidermal growth factor receptor
